# Severe anaemia in people with HIV: demographic, clinical and renal correlates

**DOI:** 10.1186/s13104-025-07100-x

**Published:** 2025-02-03

**Authors:** Kingsley Kamvuma, Sody Munsaka, Sepiso K. Masenga, John Amos Mulemena, Christopher Newton Phiri, Michelo Miyoba, Benson M. Hamooya

**Affiliations:** 1 HAND Research Group, School of Medicine and Health Sciences, Department of Pathology and Microbiology, Mulungush University, Livingstone campus, Livingstone, Zambia; 2 Department of Biomedical Sciences, University of Zambia of Health Science and Medicine, Lusaka, Zambia

**Keywords:** Severe anaemia, Renal insufficiency, PLWH, ART

## Abstract

**Objective:**

This study aimed to investigate the sociodemographic, clinical and renal correlates associated with severe anaemia among people with HIV.

**Methods:**

We conducted a cross-sectional analysis of people with HIV on antiretroviral therapy (ART) for at least 6 months, stratified by anaemia status. Anaemia was defined based on the World Health Organisation (WHO) classification, as haemoglobin concentration lower than normal i.e. <12 g/dl in females and < 13 g/dl in males and the primary outcome, severe anaemia, as a haemoglobin level below 8 g/dl according to the World Health Organisation.

**Results:**

The study comprised 372 participants receiving ART, of whom 236 (63.4%) were females. The mean age ± SD of the participants was 44.8 ± 12.4 years. The overall prevalence of severe anaemia was 7.8% (95% CI: 0.053–0.111). In multivariable logistic regression analysis, factors significantly associated with severe anaemia were female sex (Adjusted Odds Ratio (AOR: 14.3, 95% CI: 2.14–126.6), albumin (AOR: 0.93 95% CI: 0.88–0.98) and creatinine levels (AOR: 1.01 95% CI: 1.00-1.03).

## Introduction

Severe anaemia is a serious comorbidity of HIV which can rapidly complicate the quality of life and disease prognosis [1, 2]. The risk of mortality increases significantly with more severe forms of anaemia [3–5]. Even with effective antiretroviral therapy (ART), the challenge of severe anaemia in People living with HIV (PLWH) persists [6–8], highlighting the need for further inquiry. While the direct impact of HIV on haematopoiesis is well-documented, other contributing factors such as opportunistic infections, nutritional deficiencies, and prolonged HIV medications may further complicate the pathophysiology of anaemia in PLWH [9, 10]. Furthermore, socio-economic factors and limited healthcare access can exacerbate the risk of severe anaemia in PLWH.

Reduced erythropoietin production, impaired iron utilization, and inflammation contribute to anaemia in these patients. Investigating the mechanisms associated with severe anaemia is essential for targeted interventions [11]. Renal insufficiency, a common comorbidity in HIV, can lead to reduced erythropoietin production and subsequent anaemia, further complicating the clinical management. Despite the recognition of this clinical entity, there remains a paucity of literature specifically addressing severe anaemia among treated PLWH. This study aimed to investigate the correlates of severe anaemia in PLWH.

## Materials and methods

### Study design and setting

This was a cross-sectional study among adult PLWH who had been on ART for at least 6 months or more, between 1st September 2023 and 26th February 2024 at Livingstone University Teaching Hospital (LUTH) a referral hospital in the southern part of Zambia.

### Eligibility criteria

We recruited adult PLWH aged 18 years and above who had been receiving ART for ≥ 6 months. In this study, pregnant women, participants with excessive menstrual bleeding and malignant neoplasms were excluded.

### Sample size calculation

PLWH on treatment is 3880 at LUTH ART Clinic, we hypothesized a maximum percentage prevalence of 50% and 95% confidence level, and we used an online OpenEpi software [12]. We accounted for missing data by adding a 30% contingency, the final determined sample size was 372.

### Study variables and definitions

Sociodemographic data, including age, sex, blood pressure, alcohol use and physical exercise were collected from participants and health records (SmartCare and patient files) using a structured questionnaire and data collection form.

Kidney function (eGFR) was assessed according to the simplified version of the Modification of Diet in Renal Disease (MDRD) study equation: 186 × SCr(mg/dl)-1.154 × age(years)-0.203 × 0.742 (if female) × 1.210 (as our population are Africans) [13]. Staging of renal function was based on the National Kidney Foundation Disease Outcomes Quality Initiative (K/DOQI) classification [14]. Normal renal function was defined as normal or increased eGFR (eGFR ≥ 90 ml/min/1.73 m^2^) [14]. Mild, moderate, and severe renal impairment were defined as eGFR 60–89.9, 30–59.9 and 15–29.9 ml/min/1.73 m2, respectively. Impaired renal function was defined as eGFR < 60 ml/min/1.73 m2[12]. Furthermore, anaemia was defined based on the World Health Organisation (WHO) classification, as haemoglobin concentration lower than normal i.e. <12 g/dl in females and < 13 g/dl in males. Further sub-classified the anaemia as mild (11–12.9 g/dl for males and 11–11.9 g/dl for females), moderate (8–10.9 g/dl) and severe (< 7.9 g/dl) [15].

#### Primary outcome

Severe anaemia was defined as a Haemoglobin level below 8 g/d based on the World Health Organisation criteria [15].

### Blood samples and measurements

Viral load and CD4 lymphocyte count samples were collected in ethylenediaminetetraacetic acid (EDTA) containers; Becton Dickson (BD) flow cytometer was used to analyse total lymphocyte and CD4 lymphocyte count, while viral load was analysed using Ampliprep/Taqman 96 PCR analyzer. Haemoglobin (Hgb) and full blood count values were determined using the haematology analyzer Sysmex XT2000 (Abbott Laboratories Diagnostics Division, USA) and CD4 + T cells were assayed using the BD FACSCOUNT system (Becton Dickenson and Company, California, USA). Biochemical analyses were done on a Pentra C200 and HumaStar 80 clinical chemistry analyzer (Human Diagnostics, Germany) using kits supplied by the manufacturer.

### ART regimens

Protease inhibitor regimens consist of either lopinavir/ritonavir (LPV/r) or atazanavir/ritonavir (ATV/r) combined with one of the following NRTI combinations: ABC/XTC, zidovudine/XTC (AZT/XTC), or TDF/XTC NNRTI regimens include efavirenz (EFV) or nevirapine (NVP) combined with either Abacavir and lamivudine/emtricitabine (ABC/XTC) or tenofovir disoproxil fumarate and lamivudine/emtricitabine (TDF/XTC). An INSTI regimen comprises Dolutegravir (DTG) combined with TDF/lamivudine (TDF/3TC).

### Statistical analysis

SPSS version 22 was used for statistical analysis. Categorical data were summarized with frequencies and proportions, and continuous variables with medians and interquartile ranges (IQR) due to non-normal distribution, confirmed via Q-Q plots and the Shapiro-Wilk test. The Pearson chi-square test assessed significant associations between categorical variables. We compared the groups using one-way ANOVA for normally distributed variables, or otherwise using the Kruskal-Wallis test for variables that were not normally distributed. Logistic regression (univariable and multivariable) was utilized to estimate factors associated with severe anaemia. Covariates included in the final model were selected based on published evidence and variables found to be statistically significant in univariable analysis.

### Ethical considerations

Ethical approval for the study was obtained on 7th August 2023 from the University of Zambia Biomedical Research Ethics Committee (UNZABREC- REF. REF. NO. 4062 − 2023). The purpose of the study was explained to all the participants in a language familiar to them, and they provided written informed consent after agreeing to take part in the study. The research was conducted in accordance with the Declaration of Helsinki on ethical principles for medical research involving human subjects [16] and relevant local regulations and guidelines.

## Results

### Sociodemographic and clinical characteristics of the study participants

The study comprised 372 participants receiving ART, of whom 236 (63.4%) were females. The mean age ± SD of the participants was 44.8 ± 12.4 years, with a range of 18 to 79 years. The overall prevalence of severe anaemia was 7.8% (95% CI: 5.3–11.1). Furthermore, a significant proportion of participants reported alcohol consumption (210, 56.8%). Additionally, 12.2% (45/370) of participants were hypertensive, while 28.4% (105 out of 370) engaged in regular physical exercises. In terms of ART regimens, the majority of patients were on NNRTI-based regimens (*n* = 258, 69.7%), followed by INSTI-based regimens (*n* = 65, 17.6%).

### Relationship of anaemia status with demographic and clinical factors

**Fig. 1:**
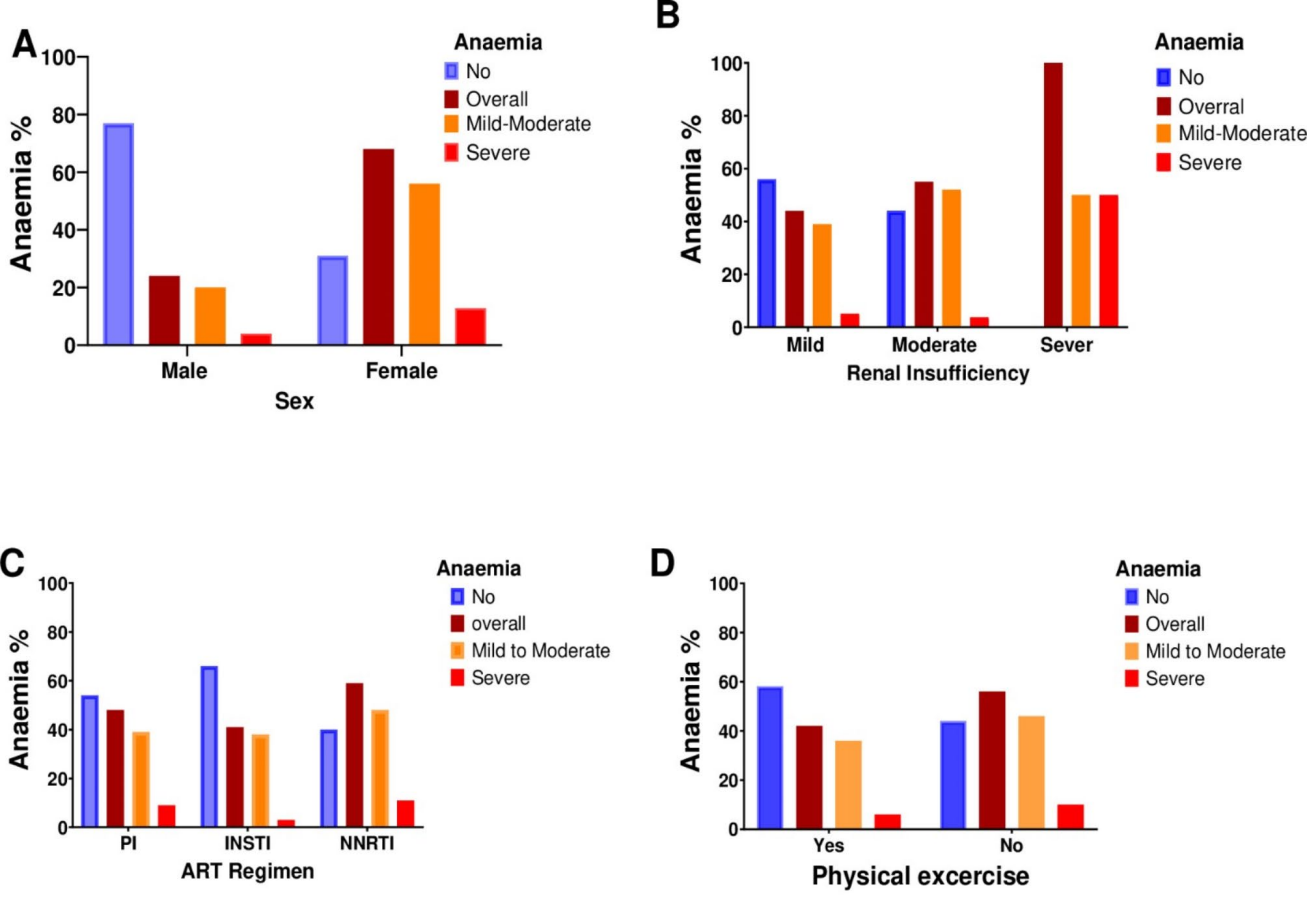
Prevalence of anaemia and Severity based on Sex, Renal insufficiency, ART Regimens and Physical exercise. ART Antiretroviral therapy, PI Protease Inhibitor, INSTI Intergrase Strand Transfer Inhibitor, Non Nucleoside Reverse Transcriptase Inhibitor, *P* < 0.05

The baseline characteristics of patients stratified by anaemia status among PLWH. It reveals significant associations between anaemia severity and various demographic, clinical, and lifestyle factors. A higher prevalence of anaemia is observed among females compared to males (Fig. 1A). Additionally, significant differences based on ART regimens are evident across anaemia categories (Fig. 1C). Lifestyle factors such as physical exercise (Fig. 1D) and alcohol consumption also demonstrate significant associations with anaemia levels. Furthermore, differences in clinical parameters including albumin levels, CD4 counts, creatinine levels, and duration of ART highlighted the significant differences with the anaemia categories in treated HIV patients, Table 1.

**Table 1:** Baseline characteristics of the patients according to haemoglobin levels he patients according to haemoglobin levels

Variable	Total	Anaemia Status			*P*-value
		No Anaemia	Mild-Mod	Severe	
Age Mean SD	372	44.7 ± 12.1	45.4 ± 12.5	43.7 ± 13.9	0.464
Sex					**< 0.001**
*Male*	136	104 (76.5)	27 (19.9)	5 (3.7)	
*Female*	234	72 (30.8)	132 (56.4)	30 (12.8)	
*Total*	370	176	159	35	
Regimen	*n* = 369				**0.016**
*PI*	46	25 (54.3)	18 (39.1)	4 (8.7)	
*INSTI*	65	43 (66.2)	20 (30.8)	2 (3.1)	
*NNRTI*	258	108 (40.3)	121 (47.5)	29 (11.2)	
*Total*	369	176	159	35	
Physical exercise					**0.035**
*Yes*	105	61 (58.1)	38 (36.2)	6 (5.7)	
*No*	261	114 (41.1)	120 (46.7)	27 (10.3)	
*Total*	366	175	158	33	
Alcohol *n* = 366					**0.002**
*Yes*	210	117 (55.7)	76 (36.2)	17 (8.1)	
*No*	159	59 (37.1)	82 (51.6)	18 (11.3)	
*total*	369	176	158	35	
Renal Insufficiency	*n* = 367				**< 0.001**
*Normal*	164	64 (39)	78 (47.6)	22 (13.4)	
*Mild*	174	98 (56.3)	70 (40.2)	6 (3.4)	
*Moderate*	27	12 (44.4)	15 (55.6)	0 (0)	
*Severe*	2	0 (0)	1 (50)	1 (50)	
*Total*	367	174	164	29	
Alt U/L	361	15 (13, 27)	19.7 (16, 26)	16.9 (12, 26)	0.16
Albumin g/dl	288	42.4 (39, 46)	41.9 (37, 44)	40.7 (34, 43)	**0.009**
Ast U/L	365	31.1 (26, 40)	30.4 (24, 43)	30.8 (23, 40)	0.92
CD4 + t (cells/mL) m(IQR)	371	611 (394, 806)	665 (363, 906)	453 (325, 805)	**0.009**
Creatinine mg/dL	369	81.6 (70, 97)	76.8 (67, 90)	70.1 (66, 80)	**0.002**
Duration ART mo, m(IQR)	372	61 (8.5, 65.5)	66.5(31, 71)	64 (30.5, 73)	**0.02**
VL(copies/mL)	372	0 (0, 20190)	0 (0, 84)	20 (0, 91)	0.92
BMI kg/m2, m(IQR)	372	21.7 (18, 28)	22.1 (19, 24)	22.6 (20, 27)	0.77
MAP mmHg	372	87.2 (80, 99)	82.8 (79, 89)	84.3 (77, 92)	**< 0.001**
Weight m, IQR	372	55 (49, 70)	58.5 (52, 64)	63 (52, 69)	**0.014**
eGFR ml/min	369	80 (62, 92)	90 (80, 103)	99 (90, 116)	**0.008**
MCV fl.	371	98 (92.5, 103)	95.7(89, 101)	91 (71.8, 96.5)	0.051

Values are presented as mean ± standard deviation for the continuous variables and frequency (percentage) for the categorical variables

eGFR: estimated glomerular filtration rate, MCV: mean corpuscular volume, FBS: Fasting blood sugar, VL: Viral load, ESR: erythrocyte sedimentation rate, NNRTI = non-nucleoside/nucleotide reverse transcriptase inhibitor (EFV = efavirenz and NVP = Nevirapine), PI = protease nhibitor (LPV/r = lopinavir/ritonavir and ATV/r = atazanavir/ritonavir), INSTI = integrase strand transfer inhibitor (DTG = dolutegravir), NRTI = nucleotide reverse transcriptase inhibitor

### Renal insufficiency and anaemia prevalence in PLWH

In our study, we observed varying degrees of renal insufficiency among the participants. Specifically, 166 individuals (44.8%) had normal kidney function, 175 (47.4%) had mild insufficiency, 27 (7.3%) had moderate insufficiency, and 2 (0.5%) had severe insufficiency based on their estimated glomerular filtration rate (eGFR) values. As renal function declines, from mild renal insufficiency to severe renal insufficiency, there is a corresponding increase in the prevalence of anaemia (Fig. 1B). Severe anaemia was more prevalent in patients with severe renal insufficiency 50%, followed by mild renal insufficiency 5% (Fig. 1B). The prevalence of overall anaemia among participants with impaired renal function (eGFR < 60 mL/min/1.73 m^2^) was higher (44.8%) than those without impaired renal function (35.6%, *p* = 0.32).

### Factors associated with severe anaemia

In the unadjusted analysis (Table 2), several factors were found to be associated with severe anaemia in PLWH. Female participants had significantly higher odds of severe anaemia compared to males (OR = 3.95, 95% CI:1.34–11.59, *p* = 0.013). Lower albumin levels were also associated with an increased risk of severe anaemia (OR = 0.95, 95% CI: 0.91–0.99, *p* = 0.024). Mean arterial pressure (MAP) showed a marginal association, with lower MAP increasing the odds of severe anaemia (OR = 0.96, 95% CI: 0.93–0.99, *p* = 0.023). Estimated glomerular filtration rate (OR = 1.01, 95% CI: 0.99–1.02, *p* = 0.25) was also included in the univariate model, but it did not emerge as a significant factor. Other variables, including age, viral load, CD4 + count, BMI and creatinine, did not show significant associations in the unadjusted analysis.

**Table 2:** Factors associated with severe anaemia

Variables	Unadjusted analysis			Adjusted analysis		
	OR	95%CI	*P* value	AOR	95%CI	*P*-value
**Age** Mean, SD	0.99	0.96–1.02	0.57	0.98	0.94–1.01	0.195
**Sex**						
*Male*	1					
*Female*	3.947	1.34–11.59	**0.013**	16.45	2.14–126.50	**0.007**
Albumin g/dL	0.949	0.91–0.99	**0.024**	0.93	0.88–0.98	**0.014**
Creatinine mg/dL	1.001	0.99–1.01	0.878	1.01	1.00-1.03	**0.031**
VL copies/mL	1.00	1.00–1.00	0.60	1.00	1.00–1.00	0.891
CD4 + count cells/mL	0.99	0.99-1.0	0.14	0.99	0.99-1.00	0.241
BMI kg/m2	0.96	0.88–1.04	0.29	0.98	0.86–1.10	0.697
MAP mmHg	0.96	0.93–0.99	**0.023**	0.97	0.93–1.01	0.150
eGFR ml/min	1.01	0.99–1.02	0.25			

OR; odds ratio, CI; confidence interval, AOR; adjusted odds ration SD; Standard deviation; VL; Viral load; BMI; Body Mass Index, MAP; Mean Arterial Pressure, CD4; Cluster of differentiation, eGFR; Estimated Glomerular Filtration Rate

In the adjusted analysis (Table 2), Female sex remained a significant predictor of severe anaemia, with the odds increasing substantially after adjustment (AOR = 16.45, 95% CI: 2.14–126.50, *p* = 0.007). Lower albumin levels continued to show a significant association with severe anaemia (AOR = 0.93, 95% CI: 0.88–0.98, *p* = 0.014), while creatinine gained significance after adjusting for other variables (AOR = 1.01, 95% CI: 1.00–1.03, *p* = 0.031). Other variables such as age, viral load, CD4 + count, BMI, and MAP did not show significant associations after adjustment.

## Discussion

This study sought to evaluate the sociodemographic, clinical, and renal factors associated with severe anaemia in PLWH. In this study, we observed an 7.8% prevalence of severe anaemia. Factors significantly associated with severe anaemia included female sex, albumin levels, and creatinine levels. Severe anaemia complicates the management of PLWH, presenting multifaceted challenges to clinicians especially when associated with renal insufficiency. Our findings show that as renal function declines from mild to severe renal insufficiency, there is a corresponding increase in the prevalence and severity of anaemia. Similar with findings elsewhwere, our results suggest that HIV infection and renal insufficiency interact to exacerbate anaemia and its severity [13, 17]. Females exhibited significantly higher odds compared to males, consistent with previous studies demonstrating sex-based disparities in anaemia prevalence [18–20].

We also found that low Albumin is associated with severe anaemia in PLWH. Albumin is a marker of nutritional status, and transporting essential nutrients, including vitamins and minerals, which are necessary for erythropoiesis [21]. Low albumin levels may indicate increased catabolism and malnutrition, which is a known risk factor for anaemia [22]. Moreover, albumin serves is a negative acute-phase reactant, as its levels decrease during inflammatory states, which can suppress erythropoiesis [23–25]. Additionally, higher creatinine levels were significantly associated with severe anaemia in our study [26]. Elevated creatinine reflects impaired renal function contributing to the prevalence of anaemia [27]. Our study shows that renal insufficiency is prevalent among PLWH, can impair erythropoietin production subsequently hindering erythropoiesis and causing anaemia [28, 29].

Despite the insights provided by this study, several limitations warrant consideration. The cross-sectional design precludes causal inference. In addition, the low number of the outcome variable (severe anaemia) may have resulted in wider confidence intervals, reducing the precision of the estimates in the regression analysis.

In conclusion, severe anaemia represents a significant challenge in the management of HIV-infected individuals. Factors such as female sex and creatinine levels, and decreased albumin levels emerged as significant factors associated with severe anaemia, highlighting the multifactorial nature of this complication. Screening for anaemia and renal function should be integrated into routine HIV care, to improve patient outcomes.

## Data Availability

No datasets were generated or analysed during the current study.

## References

[1] Fiseha T, Tamir Z, Seid A, Demsiss W. Prevalence of anemia in renal insufficiency among HIV infected patients initiating ART at a hospital in Northeast Ethiopia. BMC Hematol. 2017;17(1):1. 28116101 10.1186/s12878-017-0071-2 PMC5240406

[2] van Haalen H, Jackson J, Spinowitz B, Milligan G, Moon R. Impact of chronic kidney disease and anemia on health-related quality of life and work productivity: analysis of multinational real-world data. BMC Nephrol. 2020;21(1):88. 32143582 10.1186/s12882-020-01746-4 PMC7060645

[3] Volberding PA, Levine AM, Dieterich D, Mildvan D, Mitsuyasu R, Saag M, et al. Anemia in HIV infection: clinical impact and evidence-based management strategies. Clin Infect Dis. 2004;38(10):1454–63. 15156485 10.1086/383031

[4] Portolés J, Gorriz JL, Rubio E, de Alvaro F, García F, Alvarez-Chivas V, et al. The development of anemia is associated to poor prognosis in NKF/KDOQI stage 3 chronic kidney disease. BMC Nephrol. 2013;14(1):2. 23295149 10.1186/1471-2369-14-2 PMC3623844

[5] Abioye AI, Andersen CT, Sudfeld CR, Fawzi WW, Anemia. Iron Status, and HIV: a systematic review of the evidence. Adv Nutr. 2020;11(5):1334–63. 32383731 10.1093/advances/nmaa037 PMC7490171

[6] Kaboré NF, Poda A, Zoungrana J, Da O, Ciaffi L, Semdé A, et al. Chronic kidney disease and HIV in the era of antiretroviral treatment: findings from a 10-year cohort study in a west African setting. BMC Nephrol. 2019;20(1):155. 31064340 10.1186/s12882-019-1335-9 PMC6505177

[7] Wei L, Zhao Y, Gan X, Zhao D, Wu Y, Dou Z, et al. The burden of anemia among Chinese HIV-infected patients following the initiation of antiretroviral therapy in the treat-all era: a nationwide cohort study. BMC Infect Dis. 2023;23(1):704. 37858044 10.1186/s12879-023-08675-1 PMC10588238

[8] Izzedine H, Baumelou A, Deray G. Acute renal failure in HIV patients. Nephrol Dial Transpl. 2007;22(10):2757–62. 10.1093/ndt/gfm404 17595186

[9] Akkina R. New insights into HIV impact on hematopoiesis. Blood. 2013;122(13):2144–6. 24072846 10.1182/blood-2013-08-518274

[10] Redig AJ, Berliner N. Pathogenesis and clinical implications of HIV-related anemia in 2013. Hematol Am Soc Hematol Educ Program. 2013;2013:377–81. 10.1182/asheducation-2013.1.377 24319207

[11] Shi R, Chen X, Lin H, Ding Y, He N. Incidence of impaired kidney function among people with HIV: a systematic review and meta-analysis. BMC Nephrol. 2022;23(1):107. 35300612 10.1186/s12882-022-02721-x PMC8932163

[12] Sullivan KM, Dean A, Soe MM. OpenEpi: a web-based epidemiologic and statistical calculator for public health. Public Health Rep Wash DC 1974. 2009;124(3):471–4. 10.1177/003335490912400320 PMC2663701 19445426

[13] Fiseha T, Mengesha T, Girma R, Kebede E, Gebreweld A. Estimation of renal function in adult outpatients with normal serum creatinine. BMC Res Notes. 2019;12:462. 31358035 10.1186/s13104-019-4487-6 PMC6664564

[14] National Kidney Foundation. K/DOQI clinical practice guidelines for chronic kidney disease: evaluation, classification, and stratification. Am J Kidney Dis off J Natl Kidney Found. 2002;39(2 Suppl 1):S1–266. 11904577

[15] Haemoglobin concentrations for the diagnosis of. anaemia and assessment of severity [Internet]. [cited 2024 Mar 28]. Available from: https://www.who.int/publications-detail-redirect/WHO-NMH-NHD-MNM-11.1

[16] WMA - The World Medical Association-WMA Declaration of Helsinki. – Ethical Principles for Medical Research Involving Human Subjects [Internet]. [cited 2024 May 21]. Available from: https://www.wma.net/policies-post/wma-declaration-of-helsinki-ethical-principles-for-medical-research-involving-human-subjects/

[17] Dickson M, Mody SH, Bookhart B, Zilberberg M. Kidney function and Anemia prevalence in patients with HIV infection. Blood. 2005;106(11):2259.15941905

[18] Harding BN, Whitney BM, Nance RM, Ruderman SA, Crane HM, Burkholder G, et al. Anemia risk factors among people living with HIV across the United States in the current treatment era: a clinical cohort study. BMC Infect Dis. 2020;20(1):238. 32197585 10.1186/s12879-020-04958-z PMC7085166

[19] Zhang N, Li X, Ma L, Liang Y, Liu X, Zhou F. Prevalence Trends of Anemia Impairment in adolescents and young adults with HIV/AIDS. Blood. 2023;142(Supplement 1):5192. 10.1186/s12889-024-18730-4 PMC11092044 38741063

[20] Ciccacci F, Orlando S, Sagno JB, Kamponda M, Gondwe J, Lunghi R, et al. Evaluation of nutritional conditions, haemoglobin levels, retention in care and viral suppression in a cohort of HIV-infected Malawian adolescents undergoing a one-year tailored intervention within the diseases Relief through Excellence and Advanced Means programme. South Afr J Child Health. 2020;14(4):228–32.

[21] Gjyzari A, Idrizi A, Gjata M, Ylli D, Gjyzari I, Barbullushi M, SP398malnutrition risk and low serum albumin level in maintenance hemodialysis PATIENTS. Nephrol Dial Transpl. 2018;33(suppl1):i480–1.

[22] Patil R, Raghuwanshi U. Serum protein, albumin, globulin levels, and A/G ratio in HIV positive patients. Biomed Pharmacol J. 2015;2(2):321–5.

[23] Mirsaeidi M, Omar HR, Sweiss N. Hypoalbuminemia is related to inflammation rather than malnutrition in sarcoidosis. Eur J Intern Med. 2018;53:e14–6. 29703691 10.1016/j.ejim.2018.04.016

[24] Tsirpanlis G, Bagos P, Ioannou D, Bleta A, Marinou I, Lagouranis A, et al. Serum albumin: a late-reacting negative acute-phase protein in clinically evident inflammation in dialysis patients. Nephrol Dial Transpl. 2005;20(3):658–60. 10.1093/ndt/gfh663 15795948

[25] Komáromi A, Hammarkvist F, Rooyackers O, Wernerman J, Norberg Å. Albumin synthesis in States of inflammation. Intensive Care Med Exp. 2015;3(1):A42.

[26] Norris KC, Smoyer KE, Rolland C, Van der Vaart J, Grubb EB. Albuminuria, serum creatinine, and estimated glomerular filtration rate as predictors of cardio-renal outcomes in patients with type 2 diabetes mellitus and kidney disease: a systematic literature review. BMC Nephrol. 2018;19(1):36. 29426298 10.1186/s12882-018-0821-9 PMC5807748

[27] Cusumano AM, Rosa Diez G, Tzanno-Martins C, Editorial. Glomerular filtration rate in Chronic Kidney Disease. Front Med [Internet]. 2023 Jan 10 [cited 2024 Mar 25];9. Available from: https://www.frontiersin.org/articles/10.3389/fmed.2022.1105384 10.3389/fmed.2022.1105384 PMC9872106 36703887

[28] Peng L, He Y, Zhang J, Hong D, Li G. Erythropoietin and iron for anemia in HIV-infected patients undergoing maintenance hemodialysis in China: a cross-sectional study. BMC Nephrol. 2022;23:60. 35135490 10.1186/s12882-022-02693-y PMC8827246

[29] Röling J, Schmid H, Fischereder M, Draenert R, Goebel FD. HIV-Associated Renal diseases and highly active antiretroviral Therapy—Induced Nephropathy. Clin Infect Dis. 2006;42(10):1488–95. 16619164 10.1086/503566

